# 

**Published:** 1890-11-29

**Authors:** 


					, -- unuuuuwxuy many persons nave Doen starvea^nraeimnmron^ffTrruiperieucea meaiciirmeu STTIiniT
and nurses placing an nndno nutritions value on snoh preparations aaKxtract of Moat, homo-
made beef-tea, 4o., whereas had JOHNSTON'S BOVRIL beon used in their stead, CHEMISTS, GROCERS, &C.
the patients would in w/ w nino cases out of ten THRHIIGHnilT
have gained strength Vg( to battle against IHKUUbHUUI
^Ji8?eas? ?^er fnlr lIMl they were THE UNITED KINGDOM.
A /Jsi hesitation to advise you
7 JUL your clients who have mental exertions, to use Johnston's
tonic and a palatable food."?J. M. BEAUSOLIEL, M.D.
No. 218. Vol IX.] Saturday,
November 29th, 1890.
[One Penny.

				

